# Left ventricular filling pressure assessed by exercise TDI was correlated with early HFNEF in patients with non-obstructive hypertrophic cardiomyopathy

**DOI:** 10.1186/1471-2261-14-194

**Published:** 2014-12-18

**Authors:** Guodong Ma, Ming Xu, Wei Gao, Zhaoping Li, Weihong Li, Baoxia Chen, Jieli Feng, Hongyan Wang, Wenying Ma, Hui Chen, Aidong Shen, Xinheng Feng, Yongzhen Zhang

**Affiliations:** Department of Cardiology, Beijing Friendship Hospital, Capital Medical University, 100050 Beijing, China; Department of Cardiology, Peking University Third Hospital, 100191 Beijing, China; Key Laboratory of Cardiovascular Molecular Biology and Regulatory Peptides, Ministry of Health, 100191 Beijing, China

**Keywords:** Tissue Doppler imaging, Heart failure with normal ejection fraction, Non-obstructive hypertrophic cardiomyopathy, Cardiopulmonary exercise testing, N-terminal pro-brain natriuretic peptide

## Abstract

**Background:**

Hypertrophic cardiomyopathy (HCM) patients are more susceptible to suffer from heart failure with normal ejection fraction (HFNEF). Therefore, it is critical to evaluate the relationship between left ventricular filling pressure (LVFP) and HFNEF, even if a large proportion of HCM patients have normal LVFP at rest. The objective was to assess the correlation between exercise tissue Doppler imaging (TDI) and early HFNEF in HCM patients by treadmill exercise echocardiography combined with cardiopulmonary exercise test (CPET).

**Method:**

Twenty-seven non-obstructive HCM patients and 31 age- and gender-matched healthy volunteers were enrolled in this study. All subjects underwent treadmill exercise echocardiography combined with CPET. N-terminal pro-brain natriuretic peptide (NT-proBNP) levels were analyzed before and after exercise.

**Result:**

Five HCM patients had normal LVFP at rest and increased after exercise. For this subgroup, the relationship between minute ventilation and carbon dioxide production (VE/VCO2 slope) and NT-proBNP levels were higher compared with controls and the subgroup with normal resting and stress LVFP, but was similar to the subgroup with elevated LVFP both at rest and after exercise.

**Conclusion:**

Elevated LVFP after exercise suggested the occurrence of early HFNEF in patients with non-obstructive HCM.

## Background

Hypertrophic cardiomyopathy (HCM) is characterized by left ventricular hypertrophy and is associated with a wide array of clinical symptoms and hemodynamic abnormalities, such as left ventricular outflow obstruction, diastolic dysfunction, myocardial ischemia and mitral regurgitation. The prevalence of HCM in the United States, Japan and China is approximately of 1 per 500 adults [1–3]. HCM is a risk factor for heart failure with normal ejection fraction (HFNEF) [4, 5]. HFNEF has a poor prognosis because it is often diagnosed and treated late [4, 5]. Indeed, in the early diastolic dysfunction stage, a large proportion of HCM patients only suffer from impaired exercise tolerance without any other obvious symptoms or signs (such as dyspnea and edema) before the onset of clinical heart failure [4, 5]. Therefore, there is a need to further study the diastolic dysfunction in these patients.

Elevated left ventricular filling pressure (LVFP) is critical for HFNEF diagnosis [6]. Under exercise stress, the LVFP of HFNEF patients will increase sharply because of the increase of the left ventricular preload [7–10], while this increase is not observed in adults with normal left ventricular diastolic function [11].

Tissue Doppler imaging (TDI) has been proved valuable for assessing LVFP [12–18]. The N-terminal pro-brain natriuretic peptide (NT-proBNP) is secreted by the ventricles of the heart in response to excessive stretching of the cardiomyocytes. It is therefore considered a marker of heart damage, and can be used both for screening and prognosis of heart failure [19, 20]. NT-proBNP is usually increased in patients with left ventricular dysfunction, regardless of the presence of symptoms [21].

The aim of this study was to assess the relationship between exercise TDI and early HFNEF changes in HCM patients using treadmill exercise echocardiography combined with cardiopulmonary exercise test (CPET).

## Methods

### Study population

HCM patients were enrolled from those who visited the echocardiography laboratory of the Peking University Third Hospital between November 2010 and March 2011. Exclusion criteria were: 1) Patients who had a history of left ventricular outflow tract obstruction (LVOTO) diagnosis; 2) left ventricular ejection fraction (LVEF) <50%; 3) atrial or ventricular arrhythmia; 4) valvular disease of moderate or greater severity; or 5) pericardial diseases.

Thirty-one age- and gender-matched healthy volunteers were enrolled as controls. The protocol was approved by Peking University Institutional Review Board. All subjects provided a written informed consent before participation.

### Echocardiography

Standard 2-dimensional measurements (left ventricle diastolic and systolic dimensions, intraventricular septum (IVS), posterior wall thickness (LVPW), left atrial volume, and left ventricle outflow tract) were obtained with the patient in the supine position, before and after exercise. LV mass was calculated using the Devereux formula [22].

After obtaining the resting images from the standard parasternal and apical views, all subjects were submitted to symptoms-limited treadmill exercise (modified Bruce protocol [23]). Right after the subjects stopped exercising, echocardiography was performed in the supine position, within one minute of exercise end, using an ultrasound system (Vivid I, GE Healthcare, Waukesha, WI, USA) with a 2.5-MHz transducer. From the apical window, a 2-mm pulsed Doppler sample volume was placed at the mitral valve tip, and mitral flow velocities of 3 cardiac cycles were recorded, obtaining peak velocities of the early diastolic transmitral flow (E), of the late diastolic transmitral flow (A), and of the early diastolic lateral mitral annulus velocity (Em_lateral_) were measured by TDI using the pulsed wave Doppler mode. The filter was set to exclude high frequency signals, and the Nyquist limit was adjusted to a range of 15 to 20 cm/s. Gain and sample volume were minimized to allow for a clear tissue signal with minimal background noise. Em_lateral_ was measured from the apical 4-chamber view with a 2 mm sample volume placed at the lateral corner of the mitral annulus. These measurements were made at baseline and during recovery, in the same sequence. Measurements were recorded with simultaneous electrocardiography. All data were digitally stored.

### Cardiopulmonary exercise testing

Each subject was submitted to a symptoms-limited modified Bruce protocol [23] treadmill CPET under the supervision of a qualified exercise physiologist and a physician. Expired gases were collected continuously throughout exercise and analyzed for ventilatory volume (VE), and for oxygen (O_2_) and carbon dioxide (CO_2_) content by a professional analyzer. Expired gases were reported every 30 seconds, and were reported as peak oxygen consumption (VO_2max_, ml/kg/min), peak respiratory exchange ratio (the ratio of CO_2_ production to O_2_ consumption at peak effort), and VE/VCO_2_ slope (the slope of the increase in peak ventilation/increase in CO_2_ production throughout exercise).

Heart monitoring consisted of continuous 12-lead electrocardiography, automatic blood pressure measurements, and heart rate (HR) recordings at every stage via the electrocardiogram. Test termination criteria were: 1) patient’s request; 2) ventricular tachycardia; 3) 2 mm or more of horizontal or down sloping ST-segment depression; or 4) a drop in systolic blood pressure (SBP) of 20 mm Hg or more.

### NT-proBNP measurement

Blood samples for analysis of NTproBNP were obtained before the start of exercise and at maximal exercise. Serum NT-proBNP levels were determined using an electro-chemiluminescence immunoassay according to the manufacturer's instructions performed on a Cobas E601 (Roche Diagnostics, Basel, Switzerland).

### Statistical analysis

Normally distributed continuous variables are expressed as means ± SD. Non-normally distributed continuous variables were log-transformed to normalize their distribution for analysis. Categorical data are expressed as percentages. Variables between the study groups were compared by the Student’s t-test and by one-way analysis of variance (ANOVA). Non-parametric tests were used in cases of unequal variances. Variables at rest and peak stress within each group were compared by the Student’s t-test and categorical data were compared by the pearsonchi-square test. Differences were considered significant when the p-value was <0.05. All analyses were performed using SPSS 17.0 (SPSS Inc., Chicago, IL, USA).

## Results

### Patients' characteristics

Two hundred and sixty-eight patients were screened; 103 patients suffered from atrial fibrillation, 43 refused to participate, 39 patients could not be contacted, 12 patients suffered from left ventricular outflow tract obstruction, 35 suffered from severe mitral valve regurgitation, 5 had a hydropericardium, and 4 patients had limited physical capacities. Therefore, 27 patients were included. The clinical characteristics of HCM patients and controls are compared in Table 1. There were no differences in age, gender, body mass index (BMI), and comorbidities (hypertension and diabetes).

**Table 1:** **Clinical characteristics**

Characteristics	HCM (n = 27)	Control (n = 31)	p-value
Age (mean, yrs)	54.3 ± 12.4	49.3 ± 6.4	0.075
Female gender (n, %)	11 (42.3)	16 (51.6)	0.483
Body mass index (kg/m^2^)	25.9 ± 4.6	24.6 ± 4.3	0.224
Hypertension (n, %)	10 (38.5)	7 (22.6)	0.192
Diabetes (n, %)	2 (7.9)	1 (9.7)	0.875

Hemodynamic and echocardiographic parameters are compared in Table 2. There were no statistical differences in resting and stress HR (p = 0.621 and 0.086, resepectively) and SBP (p = 0.955 and 0.615, respectively) between the two groups. Standard echocardiography showed that there were no differences in LVEF, LVESD and LVEDD (all P > 0.05). However, the HCM patients displayed larger LAD, LAA, IVS, LVPW and LVMI (all p < 0.01) (Table 2).

**Table 2:** **Hemodynamic and echocardiographic parameters**

Variable	HCM (n = 27)	Control (n = 31)	P-value
Resting HR (beats/min)	70 ± 10	69 ± 11	0.621
Stress HR (beats/min)	110 ± 19	118 ± 16	0.086
Resting SBP (mmHg)	123 ± 13	124 ± 19	0.955
Stress SBP (mmHg)	160 ± 25	156 ± 30	0.615
LAD (mm)	34.4 ± 4.8	31.3 ± 3.9	<0.01
LAA (cm^2^)	18.6 ± 3.6	16.0 ± 2.8	<0.01
IVS (mm)	15.6 ± 4.0	8.6 ± 1.3	<0.001
LVPW (mm)	9.7 ± 2.2	8.3 ± 1.2	<0.01
LVEDD (mm)	43.7 ± 5.9	44.5 ± 6.2	0.644
LVESD (mm)	25.8 ± 5.5	27.0 ± 4.9	0.407
LVEF (%)	71.3 ± 8.8	69.4 ± 6.2	0.340
LVMI (g/m^2^)	126.5 ± 31.7	78.3 ± 21.7	<0.001
Baseline E (m/s)	0.61 ± 0.16	0.72 ± 0.15	<0.05
Stress E (m/s)	0.88 ± 0.20	0.93 ± 0.20	0.356
Baseline A (m/s)	0.68 ± 0.13	0.70 ± 0.13	0.581
Stress A (m/s)	0.99 ± 0.20	1.04 ± 0.22	0.318
Baseline E/A	0.92 ± 0.29	1.06 ± 0.29	0.070
Stress E/A	0.90 ± 0.17	0.91 ± 0.22	0.840
Baseline Sm_lateral_	9.1 ± 2.3	9.7 ± 2.0	0.262
Stress Sm_lateral_	14.3 ± 4.1	16.3 ± 3.0	<0.05
Baseline Em_lateral_	8.1 ± 2.6	11.3 ± 2.3	<0.001
Stress Em_lateral_	10.3 ± 3.2	13.3 ± 2.8	<0.001

HR: heart rate; SBP: systolic blood pressure; LAD: left anterior descending artery; LAA: left atrial appendage; IVS: intraventricular septum; LVPW: posterior wall of the left ventricle; LVEDD: left ventricular end-diastolic dimension; LVESD: left ventricular end-systolic dimension; LVEF: left ventricle ejection fraction; LVMI: left ventricular mass index; E: early diastolic transmitral flow; A: late diastolic transmitral flow; Em_lateral_: early diastolic lateral mitral annulus velocity.

### Doppler mitral inflow

The baseline peak velocity of E in the HCM group was lower than in controls (0.61 ± 0.16 vs. 0.72 ± 0.15, P < 0.05). There were no differences in other mitral inflow variables between the two groups.

### TDI parameters

Both baseline and stress Em_lateral_ of the HCM group were lower than in controls (baseline: 8.1 ± 2.6 vs. 11.3 ± 2.3, p < 0.001; 10.3 ± 3.2 vs. 13.3 ± 2.8 p <0.01). There was no difference in baseline Sm_lateral_ between the two groups (baseline: 9.1 ± 2.3 vs. 9.7 ± 2.0, p > 0.05). But stress Sm_lateral_ of the HCM group were lower than in controls (14.3 ± 4.1vs. 16.3 ± 3.0 p <0.05) (Table 2). Both baseline and exercise E/Em_lateral_ were higher in HCM patients (baseline: 8.0 ± 2.5 vs. 6.5 ± 1.5, P < 0.01; exercise: 9.1 ± 3.0 vs. 7.1 ± 1.3, P < 0.01). E/Em_lateral_ increased after exercise in HCM patients (from 8.0 ± 2.5 to 9.1 ± 3.0, P < 0.01), but not in controls (from 6.5 ± 1.5 to 7.1 ± 1.3, P = 0.085) (Table 3).

**Table 3:** **Baseline and stress E/Em**_**lateral**_

Variable	HCM (n = 27)	Control (n = 31)	P-value
Baseline E/Em_lateral_	8.0 ± 2.5	6.5 ± 1.5	<0.01
Stress E/Em_lateral_	9.1 ± 3.0	7.1 ± 1.3	<0.01
P-value	<0.01	0.085	

E: early diastolic transmitral flow; Em_lateral_: early diastolic lateral mitral annulus velocity.

### CPET parameters

For HCM patients, VO_2max_ was lower (24.3 ± 5.2 vs. 27.6 ± 3.9, P < 0.01) and VE/VCO_2_ slope was higher (28.8 ± 4.0 vs. 26.9 ± 2.7, P < 0.05) than in controls (Table 4). No LVOTO was observed in the HCM patients after CPET.

**Table 4:** **CPET parameters and NT-proBNP**

Variable	HCM (n = 27)	Control (n = 31)	P-value
VO_2_max (ml/min.kg)	24.3 ± 5.2	27.6 ± 3.9	<0.01
VE/VCO_2_ Slope	28.8 ± 4.0	26.9 ± 2.7	<0.05
Baseline NT-proBNP (pg/ml)	884	72	<0.001
Stress NT-proBNP (pg/ml)	1019	79	<0.001

VO2max: maximal oxygen consumption; VE/VCO2 slope: relationship between minute ventilation and carbon dioxide production; NT-proBNP: N-terminal pro-brain natriuretic peptide.

### NT-proBNP

In HCM patients, both baseline and exercise NT-proBNP were higher than in controls (baseline: 884 vs. 72 pg/ml, P < 0.001; exercise: 1019 vs. 79 pg/ml, P < 0.001) (Table 4).

### Subgroups comparisons

**Figure 1:**
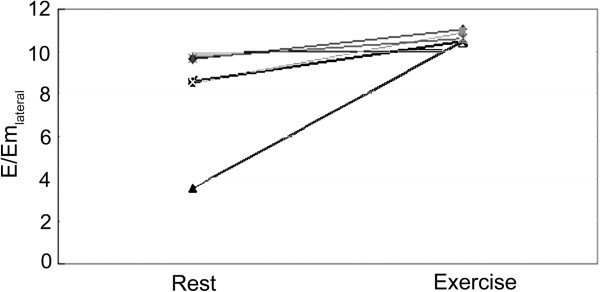
**Changes in E/Em**_**lateral**_ **after exercising in all subjects.**

**Figure 2:**
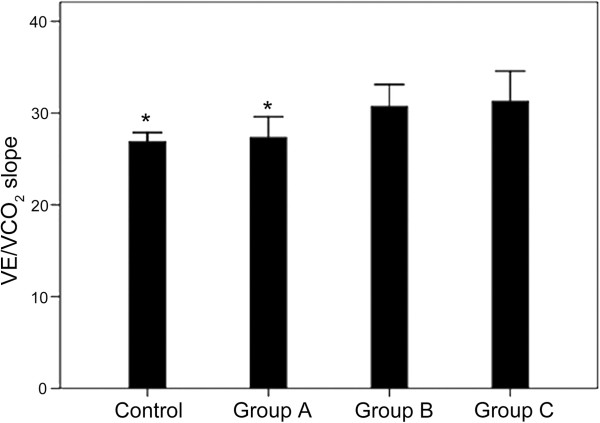
**Comparison of VE/VCO2 between the four groups. Group A**: baseline E/Em < 10 and stress E/Em < 10 (n = 17). **Group B**: baseline E/Em < 10 and stress E/Em > 10 (n = 5). Group B: baseline E/Em > 10 and stress E/Em > 10 (n = 5). Controls (n = 31). *P < 0.05 vs. group B.

**Figure 3:**
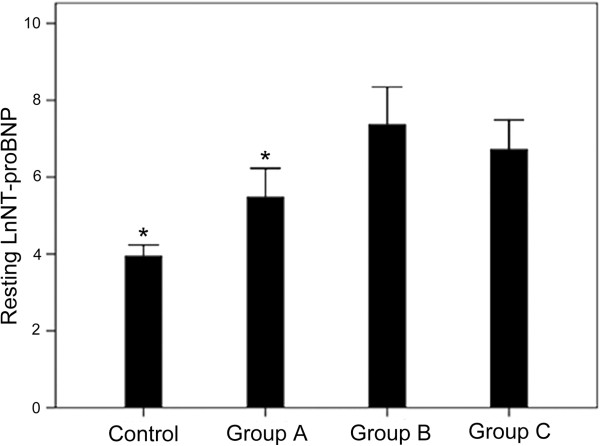
**Comparison of NT-proBNP levels at rest between the four groups. Group A**: baseline E/Em < 10 and stress E/Em < 10 (n = 17). **Group B**: baseline E/Em < 10 and stress E/Em > 10 (n = 5). **Group C**: baseline E/Em > 10 and stress E/Em > 10 (n = 5). Controls (n = 31). *P < 0.05 vs. group B.

**Figure 4:**
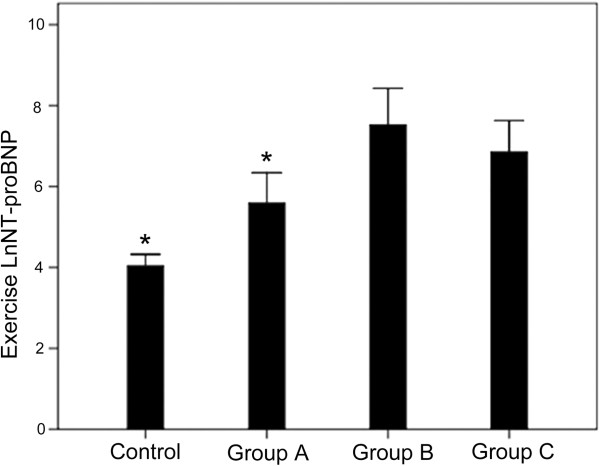
**Comparison of NT-proBNP levels after exercise between the four groups. Group A**: baseline E/Em < 10 and stress E/Em < 10 (n = 17). **Group B**: baseline E/Em < 10 and stress E/Em > 10 (n = 5). **Group C**: baseline E/Em > 10 and stress E/Em > 10 (n = 5). Controls (n = 31). *P < 0.05 vs. group B.

According to an E/Em_lateral_ above or below 10 before and after exercise, the HCM group could be divided into 3 subgroups: group A (n = 17, baseline and stress E/Em_lateral_ < 10), group B (n = 5, baseline E/Em_lateral_ < 10, and stress E/Em_lateral_ > 10), and group C (n = 5, baseline and stress E/Em_lateral_ > 10) (Table 5). In group B, the E/Em_lateral_ ratio was increased in all patients (P < 0.05) (Figure 1). There were no differences in age, BMI, LVEDD, LVESD and LVEF between the controls and the three HCM subgroups. The VE/VCO_2_ slope in group B was similar to that of group C (p > 0.05), but was higher compared with group A (p < 0.05) and controls (p < 0.05) (Figure 2). NT-proBNP in group B was similar to that of group C (p > 0.05), but was higher compared with group A (p < 0.05) and controls (p < 0.01) (Figures 3 and 4).

**Table 5:** **Parameters of the control and 3 HCM subgroups**

Variable	Control (n = 31)	Subgroup A (n = 17)	Subgroup B (n = 5)	Subgroup C (n = 5)	P-value
Age (yrs)	49.3 ± 6.4	51.1 ± 11.1	52.4 ± 12.6	66.4 ± 10.7	>0.05
BMI (Kg/m^2^)	24.6 ± 3.3	25.4 ± 4.5	25.4 ± 5.4	27.9 ± 4.7	0.391
LVMI (g/m^2^)	78.3 ± 21.7	120.1 ± 26.6	145.2 ± 51.3	127.4 ± 20.0	>0.05*
LVEDD (mm)	43.7 ± 5.9	42.7 ± 6.1	43.0 ± 6.9	48.0 ± 2.4	0.424
LVESD (mm)	25.8 ± 5.5	24.9 ± 5.2	24.4 ± 5.8	30.2 ± 4.6	0.152
LVEF (%)	69 ± 6	72 ± 9	74 ± 7	65 ± 9	0.169
Baseline E/Em_lateral_	6.5 ± 1.5	6.8 ± 1.4	8.0 ± 2.5	11.8 ± 1.2	<0.001
Stress E/Em_lateral_	7.1 ± 1.3	7.3 ± 1.3	10.5 ± 0.4	14.0 ± 2.8	<0.001
VO_2_max (ml/Kg)	27.7 ± 3.9	24.7 ± 5.0	24.8 ± 6.3	22.3 ± 5.1	<0.05
VE/VCO_2_ slope	26.9 ± 2.7	27.4 ± 4.2	30.7 ± 1.9	31.3 ± 2.6	<0.01
**Baseline LnNTproBNP**	3.9 ± 0.8	5.5 ± 1.4	7.4 ± 0.8	6.7 ± 0.6	<0.01
**Stress LnNTproBNP**	4.0 ± 0.8	5.6 ± 1.4	7.5 ± 0.7	6.9 ± 0.6	<0.01

*groups A, B and C were not different, all above the control.

BMI: body mass index; LVMI: left ventricular mass index; LVEDD: left ventricular end-diastolic dimension; LVESD: left ventricular end-systolic dimension; LVEF: left ventricle ejection fraction; VO2max: maximal oxygen consumption; VE/VCO2 slope: relationship between minute ventilation and carbon dioxide production; NT-proBNP: N-terminal pro-brain natriuretic peptide; E: early diastolic transmitral flow; Emlateral: early diastolic lateral mitral annulus velocity.

## Discussion

The main goal of this study was to assess the value of exercise TDI in early HFNEF detection in HCM patients by treadmill exercise echocardiography combined with CPET. These preliminary results showed that the higher E/Em, the worse the outcomes. E/Em may rise in a minority of patients following exercise, in whom it was normal at rest. This may indicate a rise in filling pressure during exertion and mild HFNEF. In addition, differences in stress E/Em appears to be mainly driven by the lower Em since mitral E wave peak velocity was not different between the groups.

Impaired exercise tolerance is the most common symptom in HCM patients [24]. Because diastolic dysfunction reduces the filling of the left ventricle, the heart cannot pump enough blood to satisfy the needs of the body when exercising, thus causing damage to the heart due to exercise intolerance and raised LVFP [25, 26]. However, it is also possible that the cause of exercise intolerance might be secondary to the inability of the stroke volume to increase appropriately because of abnormal sarcomeric protein causing abnormal contractility and reduced inotropic reserve [26, 27]. We observed that HCM patients had higher E/Em, VE/VCO2 slope and lower VO2max. Past studies revealed that VO2max was correlated with prognosis in heart failure patients [28]. In the present study, impaired VO2max in HCM patients may be an indicator of their bad prognosis.

Moreover, during CPET, some individuals could not reach the anaerobic threshold for some reasons. In this condition, VO2max cannot reflect the actual exercise tolerance and diastolic function. It has also been found that the VE/VCO2 slope, which was negatively correlated with prognosis, was a better indicator of prognosis in heart failure patients than VO2max, without consideration of reaching the anaerobic threshold or not [16, 29–33]. In the present study, HCM patients had higher VE/VCO2 slope than controls, suggesting that HCM patients may have worse outcomes and need to be treated earlier.

We observed that the LVFP of about 20% of our HCM patients was significantly higher immediately after exercise (baseline E/Em < 10 and stress E/Em > 10, group b), and that these patients may be diagnosed as early or latent HFNEF. As previously mentioned, the VE/VCO2 slope and NT-proBNP levels are negatively correlated with prognosis [16, 19–21, 29–33]. These two prognostic indexes were similar between groups B (i.e. latent HFNEF group) and C (i.e. clinical HFNEF), but were higher than in group A, suggesting unfavorable outcomes of early HFNEF in HCM patients. Thus, early HFNEF in HCM patients might require a particular medical attention.

Our results are supported by some previously published studies. Kitaoka et al. [34] showed that TDI after CPET was more useful than BNP levels for predicting objective capacity in HCM patients. However, they did not assess HFNEF onset. Another study by the same author showed that a high septal ratio and elevated BNP levels discriminated HCM patients who suffered from a cardiac event vs. those who did not [35]. Finally, a study suggested that TDI was useful for risk stratification of HCM patients, but BNP levels were not assessed [36].

Em measurement might have been affected by sample size, gain, filter and minimal angulation with annular motion. We selected the lateral mitral annulus, because it is easy to obtain measurements from this site from the apical window. We had experienced technician to ensure the reproducibility and to minimize the variability. Em was reduced in patients with annular calcification and mitral stenosis, and was increased in patients with moderate to severe mitral regurgitation. Therefore, these patients were not included in our study. In addition, E velocity could decrease rapidly after exercise; therefore, we measured E velocity within one minute after exercise termination. Because of the relatively small number of enrolled patients, larger studies are needed to prove the value of exercise TDI in diagnosis of HFNEF in HCM patients. However, the use of control patients enhances the reliability of our study.

## Conclusions

In conclusion, the results suggest that the LVFP of HCM patients was higher than healthy adults and that exercise TDI have a potential value for early HFNEF diagnosis in HCM patients.
